# The personality of U.S. states: Stability from 1999 to 2015

**DOI:** 10.1016/j.jrp.2016.06.022

**Published:** 2018-02-02

**Authors:** Lorien G. Elleman, David M. Condon, Sarah E. Russin, William Revelle

**Affiliations:** a Department of Psychology, Northwestern University, Evanston, IL 60208, United States; b Department of Medical Social Sciences, Northwestern University, Chicago, IL, United States

**Keywords:** Regional differences, Scale generalizability, Aggregate personality, Replication, Personality of U.S. states, Online personality assessment, Big Five, Sociodemographic

## Abstract

Researchers have shown an interest in the aggregated Big Five personality of U.S. states, but typically they have relied on scores from a single sample (Rentfrow, Gosling, & Potter, 2008). We examine the replicability of U.S. state personality scores from two studies (Rentfrow et al., 2008; Rentfrow, Gosling, Jokela, & Stillwell, 2013) across a total of seven samples, two of them new. Same-trait correlations across samples are, on average, positive for all five traits, indicating score agreement. Additionally, three traits (Conscientiousness, Neuroticism, and Openness) show strongly consistent patterns of correlations with sociodemographic variables across samples. We find rank order stability in state personality scores for a 16-year period (1999–2015).

## 1. Introduction

The beginning of the twenty-first century has seen an explosion of interest concerning geographical variation in personality within the United States. Before the modern era of the internet, there were a few studies that examined aggregate psychological differences by U.S. cities or regions (e.g., Krug & Kulhavy, 1973; Thorndike, 1939). However, with the widespread adoption of the internet in the U.S., several psychology labs have collected samples of hundreds of thousands of participants across the country via online personality assessments (e.g., Revelle, Wilt, & Rosenthal, 2010; Revelle et al., 2016; Srivastava, John, Gosling, & Potter, 2003). These samples, although not representative of the U.S. population, are more diverse than traditional methods of data collection (Gosling, Vazire, Srivastava, & John, 2004). They also have enough statistical power for analyses to detect small effects between a large number of regional groups. These online assessments typically use self-report personality assessment models based on the Big Five (Goldberg, 1990), a widely-accepted taxonomy that organizes most individual differences into five broad traits: Conscientiousness, Agreeableness, Neuroticism (sometimes referred to by its polar opposite, Emotional Stability), Openness (sometimes called Intellect), and Extraversion.

Rentfrow, Gosling, and Potter (2008) were the first to showcase one of these large samples (*n* = 619, 397) in a landmark study of U.S. regional differences of Big Five personality. Their study was the first to aggregate individual Big Five personality scores into mean state scores for all 50 states and Washington, DC. They were also first to publish the state scores for each trait, in the form of ranks and standardized scores. These published scores have proven to be of long-lasting utility to researchers interested in U.S. regional personality differences. Collecting such a massive sample is several orders of magnitude easier than in the last century, but it is by no means a trivial endeavor. Thus, many researchers have leveraged these published data to correlate U.S. state scores with state-level sociodemographics, such as chronic disease (Pesta, Bertsch, McDaniel, Mahoney, & Poznanski, 2012), obesity (McCann, 2011), income inequality (de Vries, Gosling, & Potter, 2011), and the severity of state governmental punishment (Harrington & Gelfand, 2014). Rentfrow et al. (2008) correlated state scores with sociodemographics and found some results that were congruent with their hypotheses (e.g., Openness was positively related to liberal values) and some that were unexpected (e.g., Extraversion was positively related to murder per capita).

Studies that have used the state scores data from Rentfrow et al. (2008) have assumed that these state scores were representative of the actual personality scores of the states’ residents. For example, these studies assumed that the Extraversion score for Oklahoma accurately represented the mean Extraversion score of all Oklahomans. This assumption could be problematic for at least three reasons. First, these state scores, although based on many participants, are not immune to sampling bias. Idiosyncratic methods of participant selection could lead to a lack of replication in other samples. Second, even if the original scores were accurate, the personality of some states’ residents may have changed since the original study’s data were collected (1999–2005). Third, Rentfrow et al. (2008) measured personality with the Big Five Inventory (BFI; John & Srivastava, 1999). The ranks and standardized scores from Rentfrow et al. (2008) may not generalize across other measures of the Big Five. Therefore, it is useful to examine the extent to which the state-level scores of Rentfrow et al. (2008) will replicate, and to estimate the effect of disagreement attributable to differences in participant recruitment methods, change over time, and Big Five measures. Additionally, because these state scores are often reused in other studies, it is critical to determine the extent to which correlations between state personality and sociodemographics replicate in spite of differences in samples.

A study dedicated to replicating Rentfrow et al. (2008) has not yet been reported. Rentfrow, Gosling, Jokela, and Stillwell (2013) compared state scores across five samples, including the sample from Rentfrow et al. (2008). Details from this effort were brief because the focus of the paper concerned the personality profiles of broad regions of the U.S. Findings were not thoroughly discussed, and many of the results were relegated to the supplemental materials. However, their analyses indicated that “there were no clear or consistent statewide differences in any of the scale properties,” and state scores were “reliable and generalizable” (Rentfrow et al., 2013, p. 1003). The study also found that in general, the same traits correlated with the same sociodemographic variables at similar magnitudes across the five samples.

The current study used the five samples reported from Rentfrow et al. (2013) and added two new large samples. The goals of the study were as follows: One, for a point of comparison with Rentfrow et al. (2013), determine the extent to which the two replication samples were representative of U.S. states (Section 3.1). Two, across all samples, estimate the reliability of state score differences by evaluating their intraclass correlations (Section 3.2). Three, estimate the effect size of same-trait convergent correlations across all samples for each Big Five trait (Section 3.3). Within this goal, estimate the separate effects of three possible sources of attenuation: differences due to recruitment methods (Section 3.3.1), time of data collection (Section 3.3.2), and personality inventories (Section 3.3.3). Four, from these estimates, determine whether the personality of U.S. states had maintained rank order stability (i.e., relative to each other, states’ personalities did not change) from 1999 to 2015 (Section 3.3.4). And finally, determine the replicability of correlations between state-level personality scores and sociodemographic variables (Section 3.4).

## 2. Methods

### 2.1. Samples 1–5

Samples 1–5 were originally analyzed in Rentfrow et al. (2013). The samples were collected during different time periods, as part of different research projects, using different personality inventories (Table 1). All five samples had aggregate measures of personality based on Likert-type scales for the 48 contiguous states, as well as Washington, DC. In total, the samples were collected over an 11-year period (1999–2010), with a range in sample size from 18,182 to 612,140. Samples 1–4 were online personality assessments that used self-selecting participant recruitment. Sample 5 was an online assessment that used a recruitment method similar to random digit-dialing to select a representative sample of registered voters. A more detailed summary of Samples 1–5 can be found in Rentfrow et al. (2013), where each sample is referred to by the same name used in this study.

Through correspondence with Rentfrow, we received participant counts and unadjusted mean scores for states, for each sample. Other data on the five samples, such as interclass correlations, were collected from Rentfrow et al. (2013) and the supplemental materials.

### 2.2. SAPA samples

The last two samples were from the Synthetic Aperture Personality Assessment (SAPA) project, an online non-commercial personality assessment (https://sapa-project.org; Revelle et al., 2016). For their participation, participants received feedback concerning their personality. Each sample covered an approximate five-year period of time and was named for the last year in which data were collected. The SAPA2010 sample was collected from April 2006 to August 2010. The SAPA2015 sample was collected from August 2010 to December 2015 (Table 1).

#### 2.2.1. Participants

Participants were screened to ensure that entries beyond their first were not included in the analysis. Duplicate entries taken in a single internet browser session were removed. Participants who reported having previously taken the assessment also were excluded. Since this study was concerned with state-level analysis, it was also necessary to remove participants who reported not being from one of the 50 U.S. states or Washington, DC.

After these screening procedures, there were 81,538 participants in the SAPA2010 sample (70% female), and 134,858 participants in the SAPA2015 sample (66% female). The median age for the SAPA2010 and SAPA2015 samples was 23 years (*Median Absolute Deviation [MAD]* = 7.4) and 22 years (*MAD* = 5:9), respectively. Of the 81,532 SAPA2010 participants who reported their race or ethnicity, 77% were white, 8% were African American, 6% were Hispanic, 4% were Asian, 1% were Native Alaskan/American/Hawaiian, and 4% reported being “Other.” Of the 132,838 SAPA2015 participants who reported their race or ethnicity, 67% were white, 10% were African American, 9% were Hispanic, 5% were Asian, 1% were Native Alaskan/American/Hawaiian, 6% were more than one race or ethnicity, and 1% reported being “Other.”

Concerning educational attainment, 40% of the SAPA2010 sample reported being an undergraduate at the time of assessment, while 28% had attained at least a bachelor’s degree. In the SAPA2015 sample, 51% were current undergraduates, while 27% had attained at least a bachelor’s degree.

#### 2.2.2. Personality measures

Most online personality assessments give every participant the same fixed set of items. Researchers typically analyze complete cases or use mean scores to impute the small amount of missing data. The SAPA project is radically different in this regard; participants receive a random sample of items from a pool of personality, cognitive ability, and interest inventories. Although items given within an inventory are random, sampling rates of inventories differ based on research goals of the SAPA project’s collaborators (Revelle et al., 2010, 2016).

In this study, all of the personality items in the SAPA samples were from the International Personality Item Pool (IPIP; http://ipip.ori.org/), an online repository for public domain personality items and inventories (Goldberg, 1999; Goldberg et al., 2006). We assessed participants on these items using a 1–6 Likert-type scale (1 = “Very inaccurate”; 6 = “Very accurate”).

The sole Big Five personality measure in the SAPA2010 sample was the Big Five Factor Markers (BFFM), a 100-item IPIP personality inventory based on the Goldberg (1992) conception of the Big Five. On average, participants took 48 BFFM items (48% of the inventory).

In the SAPA2015 sample we examined four measures of Big Five personality. The first was the BFFM. On average, participants took 30 BFFM items (30% of the inventory). The second personality measure was the IPIP-NEO, a 300-item IPIP inventory based on the NEO-PI-R conception of the Big Five (Costa & McCrae, 1992). On average, participants took 26 IPIP-NEO items (9% of the inventory). The third personality measure was the SAPA Personality Inventory (SPI), a 75-item inventory of IPIP items in which Condon (2014) determined the “best” items for the Big Five through empirical analyses. On average, participants took 11 SPI items (15% of the inventory). The last measure in the SAPA2015 sample was the IPIP-HEXACO (Ashton, Lee, & Goldberg, 2007), a 240-item IPIP inventory based on the six-factor HEXACO framework, which adds the Honesty-Humility trait to the Big Five (Lee & Ashton, 2004). On average, participants took 26 IPIP-HEXACO items (11% of the inventory).

The BFFM was considered to be the primary measure of personality in the SAPA2015 sample because participants took the most items from the inventory, both in terms of raw number and percent of total items in the inventory. However, an analysis in Section 3.3.3 utilized all the above personality measures of the SAPA2015 sample.

In Section 3.2, two additional measures in the SAPA2015 sample were examined: the IPIP-NEO 10-item Openness facet of Liberalism, and the 60-item ICAR (International Cognitive Ability Resource) measure of cognitive ability (Condon & Revelle, 2014). On average, participants took 2 Liberalism items (20% of the inventory) and 14 ICAR items (23% of the inventory).

### 2.3. Secondary data

#### 2.3.1. U.S. Census Bureau data

In order to weight correlations and determine the representativeness of SAPA samples, four measures from the 2010 U.S. Census were used: state populations (United States Census Bureau, 2015), state populations by ethnicity (United States Census Bureau, 2014), state populations by age (United States Census Bureau, 2016) and state populations by adult education (United States Census Bureau, 2016).

#### 2.3.2. Sociodemographic measures

Thirteen state-level sociodemographic measures were selected to cover a similar breadth of criteria as reported in previous studies (Rentfrow et al., 2008, 2013). All sociodemographic measures analyzed were either per capita or percentage of state residents. There were two measures of physical health in 2008: cancer deaths (American Cancer Society, 2008) and heart disease deaths (Miniño, Murphy, & Xu, 2011); two measures of crime in 2008: violent crime and property crime (Federal Bureau of Investigation, 2009); four measures concerning adult employment by job fields in 2009: “arts, design, entertainment, sports, and media,” “business and financial,” “computer and mathematical science,” and “healthcare practitioner and technical” (Bureau of Labor Statistics, 2009); one measure of innovation: the number of patents issued in 2008 (United States Patent & Trademark Office, 2009); two measures regarding beliefs in 2008: self-identified political liberals and people who responded affirmatively to the question, “Is religion an important part of your daily life?” (Gallup, 2014); and two measures of well-being in 2013: an index of overall well-being and self-reported community recognition in the past 12 months (Gallup, 2016).

## 3. Results

Individual personality scores in the SAPA samples were calculated using the simple mean of the observed items. State personality scores were then found by aggregating individual personality scores. State-level scores for the SAPA2010 and SAPA2015 samples, as well as a sample that combines the two, are available in Tables 6–8 of the supplemental materials. Each mean of correlations was calculated by converting correlations to *z*-scores, determining the mean, and transforming the mean *z*-score back into a correlation. Analyses were performed in the *psych* (Revelle, 2016) package and displayed using the *psych* and *corrplot* (Wei, 2013) packages in the *R* statistical system (R Core Team, 2016).

Despite large individual sample sizes, aggregating personality decreased the number of “participants” to the number of states (51 in Section 3.1; 49–51 in Section 3.2; 49 in Section 3.3; and 48 in Section 3.4). In the case of 48 participants, a conventional criterion for statistical significance (*p* < .05) would require *r*s > |.28|. Multiple comparisons were analyzed, which typically requires one to set an even higher threshold for statistical significance, in order to protect against an increased probability of finding a “significant” correlation by chance. We focused on patterns and means of correlations when possible because the standard error of a mean correlation decreases with the square root of the number of correlations that go into the mean.

### 3.1. Representative analysis of SAPA samples

To determine the extent to which the SAPA samples were representative of state populations, each sample’s number of participants per state was correlated with the Census Bureau’s 2010 population estimates. The Census Bureau’s state population estimates were highly correlated with those of the SAPA2010 sample (*r* = .93) and SAPA2015 sample (*r* = .95). These results were similar to those of Rentfrow et al. (2013)

For the SAPA samples’ representativeness of ethnicity within each state, we correlated each sample’s ethnicity percentages in each state with the Census Bureau’s estimates. For example, in each sample we correlated the percentage of African American participants in each state with the Census Bureau’s estimates of African American percentage of state populations. Correlations between the SAPA2010 sample and the Census Bureau’s estimates were in descending order as follows: African American (*r* = .98), Asian (*r* = .98), white (*r* = .95), Hispanic (*r* = .94), Native American (*r* = .84), and “Other” (*r* = .7; correlated with the Census Bureau’s “two or more” category). Correlations between the SAPA2015 sample and the Census Bureau’s estimates were in descending order as follows: Hispanic (*r* = .97), Asian (*r* =.96), white (*r* = .96), African American (*r* = .93), Native American (*r* = .87), and more than one race or ethnicity (*r* = .85). These results were similar to those of Rentfrow et al. (2013).

To determine the SAPA samples’ representativeness of age within each state, states’ percentages of participants by four age groups (18–24, 25–44, 45–64, and over 64) were correlated with the Census Bureau’s population estimates. The SAPA2010 sample was not correlated with the Census Bureau’s estimates of state population for age groups 18–24, 25–44, 45–64, and over 64 (*r* =.00, −.18, .05, and .13, respectively). The SAPA2015 sample also was not correlated with the Census Bureau’s estimates (*r* = −.08, .11, .14, .11). Rentfrow et al.(2013) had similar findings in that their age groups had low or no correlations with the Census Bureau’s estimates.

For the representativeness of states’ education levels in the SAPA samples, we correlated each sample’s percent of participants over 17 years old who attained at least a high school degree with the Census Bureau’s 2010 state population estimates for the same measure. Census Bureau state high school education was not correlated with SAPA2010 sample (*r* = −.11) and was negatively correlated with the SAPA2015 sample (*r* = −.28). We performed the same analysis for participants over 24 who had attained at least a bachelor’s degree, and found that Census Bureau state college education was positively correlated with the SAPA2010 sample (*r* = .48) and the SAPA2015 sample (*r* = . 29). Thus, the SAPA samples did not appear to be representative of U.S. states in terms of high school education, but were more representative in terms of college education. Rentfrow et al. (2013) did not test for representativeness of education.

### 3.2. Reliability of state differences: individual and group variation

Intraclass correlations (ICCs) describe different variance ratios of an aggregated variable. ICC1 is often conceptualized as a measure of interrater reliability (Bliese, 2000), and it can also represent the percent of total variance in participants’ scores that is explained by group membership (Shrout & Fleiss, 1979). In the context of this study, ICC1 indicated, for each personality scale score in each sample, the percent of variance at the individual level that could be accounted for by being a resident of a state. If individuals actually differed in their personality due to state residence, ICC1 estimated the size of this effect. ICC2 is a measure of group mean reliability (Bliese, 2000). In the context of this study, ICC2 indicated, for each personality scale score in each sample, the extent to which the aggregated state scores were reliably different from one another. Given a random sample, if a new random sample were to be collected with the same average number of participants per state, ICC2 estimated the correlation between the scores of the first sample and the second sample (James, 1982). ICC2 is functionally the Spearman-Brown formula applied to ICC1 and the average number of observations in a group, k, as shown in Eq. (1):

(1) $$\text{ICC}2=\frac{k\left(\text{ICC}1\right)}{1+\left(k-1\right)\text{ICC}1}$$

Just as small effects can be “statistically significant” in large samples, a small ICC1 and a large average group size will produce a large ICC2. James (1982) suggested using ICC1 as a criterion for aggregation. It would be unrealistic to expect that most of an individual’s personality would be due to state residence. However, studying the personality of states presumes that a non-trivial amount of individual variance in personality is explained by state variance in personality.

For each of the seven samples, the mean ICC1 across the Big Five was less than one-half of one percent (*min* = .0001; *max* = .0030; Table 2).^1^ Thus, the samples consistently agreed that state residence accounted for a small amount of variance in individual personality scores. Due to the large average number of participants per state, however, the mean ICC2 across the Big Five indicated that samples had at least a fair group mean reliability ($\bar{\text{ICC}2}>.75$), except Sample 5 ($\bar{\text{ICC}2}>.52$; Table 2).

Because ICC1 values were so small, we felt it prudent to determine whether random aggregation would produce an ICC1 of a similar magnitude. If so, the result would indicate that the “reliable” group mean differences were likely attributable to sampling error. In the SAPA2015 sample, we performed a simulation (1000 iterations) in which participants were randomly assigned (without replacement) to states, keeping the number of participants per state constant. The simulation’s average ICC1 was zero (to five decimal places), and its ICC2 was slightly negative ($\bar{\text{ICC}2}>-.04$; Table 2). Thus, the observed small effect of personality aggregation at the state level did not appear to be the result simply of aggregating data.

In a small side analysis, we attempted to determine whether two additional personality constructs (a lower-level personality facet and cognitive ability) would have larger ICC1 values than the Big Five. For this test, two measures from the SAPA2015 sample were used: the IPIP-NEO Openness facet of Liberalism and the ICAR measure of cognitive ability. Compared to Openness, Liberalism is a construct more focused on political views. We predicted that state residence would account for a large amount of individual variance in Liberalism because of state-level political differences. While some evidence suggests that states differ in cognitive ability (McDaniel, 2006), comparisons of aggregation effect sizes with personality have not yet been examined. For both Liberalism and cognitive ability, ICC1 values were greater than .01 (Table 2). This amount of variance explained is typically considered to be small (and perhaps not noteworthy), but the variance explained by state residence for Liberalism (ICC1 = .0137) and cognitive ability (ICC1 = .0122) were both roughly four times larger than the average variance explained for the Big Five in the SAPA2015 sample ($\bar{\text{ICC}1}=.0030$; Table 2). It is also noteworthy that this larger ICC1 greatly reduced the average number of participants per state needed for an acceptable group mean reliability. For example, in the SAPA2015 sample, Liberalism had fewer average participants per state (*k* = 459) than the BFFM Big Five (*k* = 2577) due to the lower sampling rate of Liberalism items. However, Liberalism group mean reliability (ICC2 = .86) was on par with the Big Five ($\bar{\text{ICC}2}=.88$).

Another way to conceptualize the small effect size of ICC1 is to consider the magnitude of standard deviations and ranges for state personality scores. For example, in the SAPA2015 sample, unstandardized Extraversion scores at the individual level had a standard deviation of 1.18 (*range* = 5; Table 3). The standard deviation of state-level Extraversion scores was .08, and the range was .43 (Table 3). An example of these results can be expressed in the following statement: On a 6-point scale, an average person from Washington, DC, the most extraverted “state,” would score a 3.97, whereas an average person from Wyoming, the least extraverted state, would score a 3.54. Liberalism, on the other hand, had a state-level standard deviation three times as large as Extraversion (*SD* = .26), and a range of 1.44 (Table 3). That is, an average person from Vermont, the most liberal state, would score a 4.31, whereas an average person from Mississippi, the least liberal state, would score a 2.87. Thus, states varied on trait Liberalism substantially more than any of the Big Five.

### 3.3. Same-trait convergent correlations for state-level personality scores

**Fig. 1.**
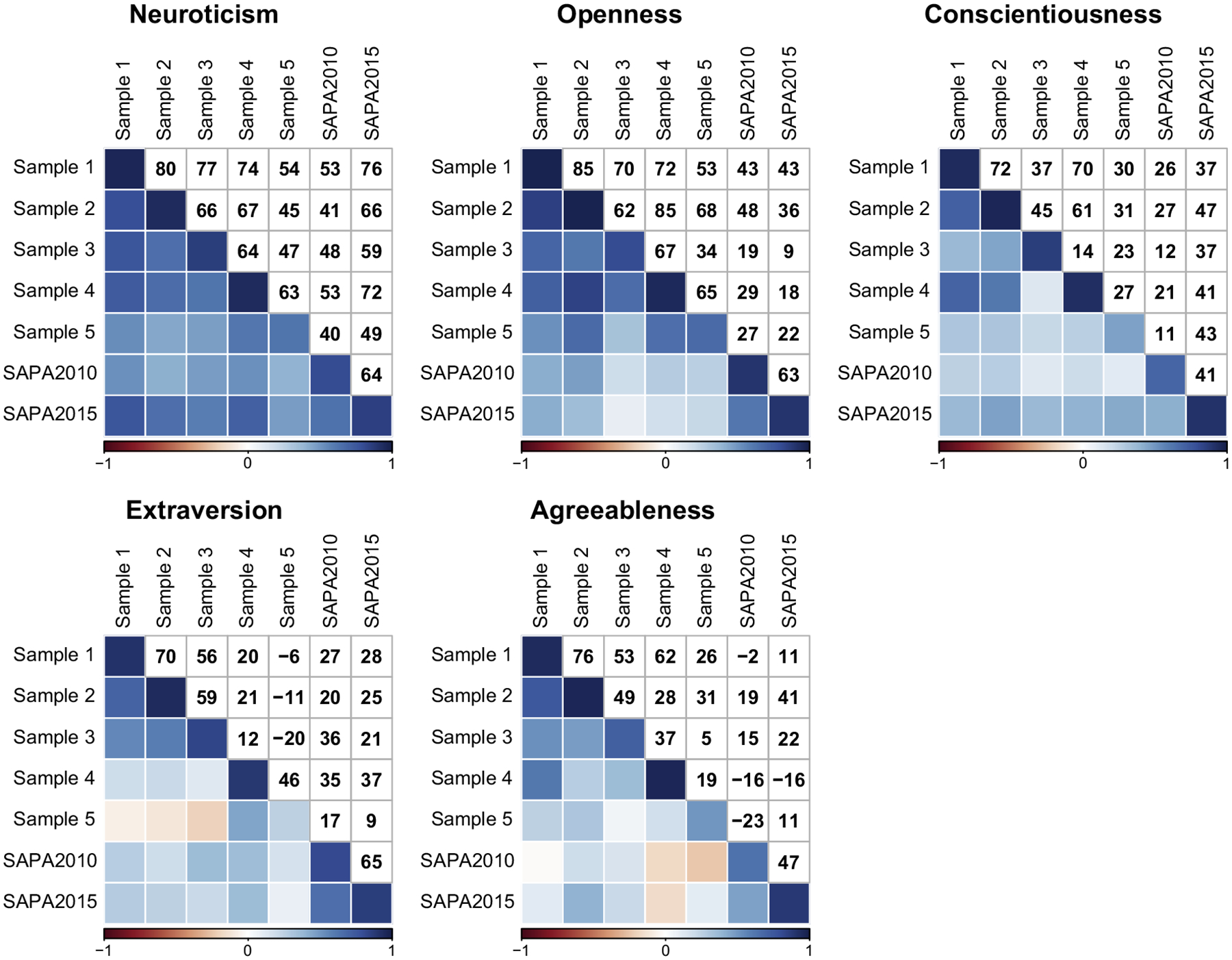
Census-weighted same-trait correlations of state-level personality, color-coded for size and sign of correlation. Upper triangle shows correlations with decimal removed. Diagonal is the group mean reliability of the trait (ICC2) for a given sample. (For interpretation of the references to color in this figure legend, the reader is referred to the web version of this article.)

To determine the magnitude of same-trait agreement across the seven samples, same-trait convergent correlations were calculated for state-level personality scores (Fig. 1). Due to some samples containing few participants per state (e.g., *min* = 29 in Sample 5), correlations were weighted by 2010 Census estimates of state populations, which were highly correlated with the number of state participants for each sample ($\bar{r}=.97$). Correlations were based on 49 state-level observations: the 48 contiguous states and Washington, DC, due to Samples 1–5 being limited to these 49. Weighted correlations adjusting for attenuation due to group mean reliability (ICC2) were also calculated, and are available in Fig. 1 of the supplemental materials.

To determine how well the SAPA samples replicated the personality scores from the five samples in Rentfrow et al. (2013), the mean same-trait correlations of Samples 1–5 were correlated with the two SAPA samples. The mean correlation across the five traits was small but positive ($\bar{r}=.31$). Same-trait correlations differed by trait; Neuroticism had the largest correlation ($\bar{r}=.57$), followed by Conscientiousness ($\bar{r}=.31$), Openness ($\bar{r}=.30$), Extraversion ($\bar{r}=.26$), and Agreeableness ($\bar{r}=.06$). Thus, Agreeableness appeared to be the only trait whose scores showed no evidence of replication in the SAPA samples.

For subsequent analyses, we treated the SAPA samples as two samples in a larger analysis of seven samples. The overall mean same-trait correlation across the seven samples was .42. Mean same-trait correlations differed by trait, but all were positive. Scores for Neuroticism ($\bar{r}=.61$) and Openness ($\bar{r}=.52$) were most consistent across the seven samples, whereas correlations for Conscientiousness ($\bar{r}=.37$), Extraversion ($\bar{r}=.29$), and Agreeableness ($\bar{r}=.26$) were smaller.

Each mean same-trait correlation was a composite of 21 correlations between pairs of the seven samples (Fig. 1). Samples differed in terms of their personality inventory, participant recruitment related to underlying research project, and the time period in which they were collected (Table 1). Any one of these differences could have led to attenuation of a same-trait correlation.^2^ We grouped correlations by these three differences to determine whether any of them were related to attenuation in same-trait correlations (Table 4). Where possible, we estimated the effect of one type of difference by only evaluating correlations whose samples differed in that one way.

#### 3.3.1. Estimating convergence between pairs of samples in adjacent time periods

This analysis required a pair of samples to be from the same research project and use the same personality inventory, but be from adjacent time periods. There were two pairs of samples that met these criteria. Sample 1 and Sample 2 both measured personality with the 44-item BFI and were part of the Gosling-Potter Internet Personality Project. The SAPA2010 and SAPA2015 samples both measured personality with the 100-item BFFM and were part of the SAPA Project. Overall convergence was positive and considerable ($\bar{r}=.68$; Table 4). On average, Openness/Intellect had the largest same-trait correlation across time ($\bar{r}=.76$) and Conscientiousness had the smallest ($\bar{r}=.59$). The SAPA samples appeared to agree less ($\bar{r}=.57$) than the Gosling-Potter samples ($\bar{r}=.77$; Table 4). This result indicated considerable rank order stability of state personality scores across adjacent time periods.

#### 3.3.2. Estimating convergence between pairs of samples in different research projects

This analysis required there to be a pair of samples from the same time period, that measured personality with the same inventory, but from different research projects. There was one pair of samples that met these criteria: Sample 3 and Sample 5. Both samples were collected during an overlapping period of time (Sample 3: 2002–2009; Sample 5: 2007–2008) and used the 10-item TIPI personality inventory. Overall same-trait convergence between Sample 3 and Sample 5 was positive but low ($\bar{r}=.19$; Table 4). There were three other samples in the same time period that used different inventories and were in different projects than Samples 3 and Sample 5: Sample 2, Sample 4, and SAPA2010. Grouping all five samples together resulted in nine sample pairs (excluding the Sample3-Sample5 pair). Overall same-trait convergence between these pairs was positive but low ($\bar{r}=.38$; Table 4). Average agreement was highest for Neuroticism/Stability ($\bar{r}=.55$) and Openness/Intellect ($\bar{r}=.56$), and lowest for Agreeableness ($\bar{r}=.18$). Because these samples measured personality with different inventories, we next estimated the attenuation of same-trait correlations associated with different personality inventories.

#### 3.3.3. Estimating convergence between different personality inventories

This analysis required a single sample to have state scores from multiple personality inventories. The SAPA2015 sample was the only sample that met this criterion. Because the IPIP-based inventories of the SAPA2015 sample had overlapping items, the correlations between scale scores at the individual level were not independent and would be inflated. Due to a concern that correlations between state scores also would show this bias, same-trait correlations across inventories were estimated with a bootstrapping method in which the state scores from two random halves of the SAPA2015 sample were correlated for 1000 iterations. Means were found across iterations. Mean same-trait correlations across inventories were moderate and positive across all traits ($\bar{r}=.68$), and of a similar magnitude to the estimated reliabilities of the inventories ($\bar{r}=.75$; Table 5). When adjusting for attenuation due to reliabilities, most same-trait correlations across inventories were .9 or larger (Table 5). Thus, the low same-trait correlations in Section 3.3.2 appeared to be related mostly to samples being from different research projects, not different personality inventories.^3^

#### 3.3.4. Estimating personality rank order stability of U.S. states over a sixteen-year period

In the previous sub-sections, we found estimates for the mean same-trait correlation when sample pairs were collected in adjacent time periods ($\bar{r}=.68$; Table 4), were part of different projects ($\bar{r}=.38$; Table 4), and used different inventories ($\bar{r}=.68$; Section 3.3.3). Sample pairs that differed on all three of these factors (i.e., adjacent time periods, different projects, and different inventories) had a mean same-trait correlation of .41 (Table 4). Furthermore, for the one pair of samples that were in time periods separated by five years, Sample 1 (1999–2005) and SAPA2015 (2010–2015), the mean same-trait correlation was .42 (Table 4). The mean same-trait correlations of samples that were part of different projects and used different inventories were virtually the same, regardless of whether the samples were collected in the same time period, in adjacent time periods, or in time periods separated by five years. Thus, state personality scores had maintained rank order stability over a sixteen-year period.

### 3.4. Replicability of state personality correlations with sociodemographics

Weighted correlations (as described in Section 3.3) between personality scores of the seven samples and the thirteen sociodemographic variables were partialled, controlling for the other four personality scores within a sample (Tables 1–5 in the supplemental materials). These partial correlations were limited to the 48 contiguous states due to limitations of the secondary data.

**Fig. 2.**
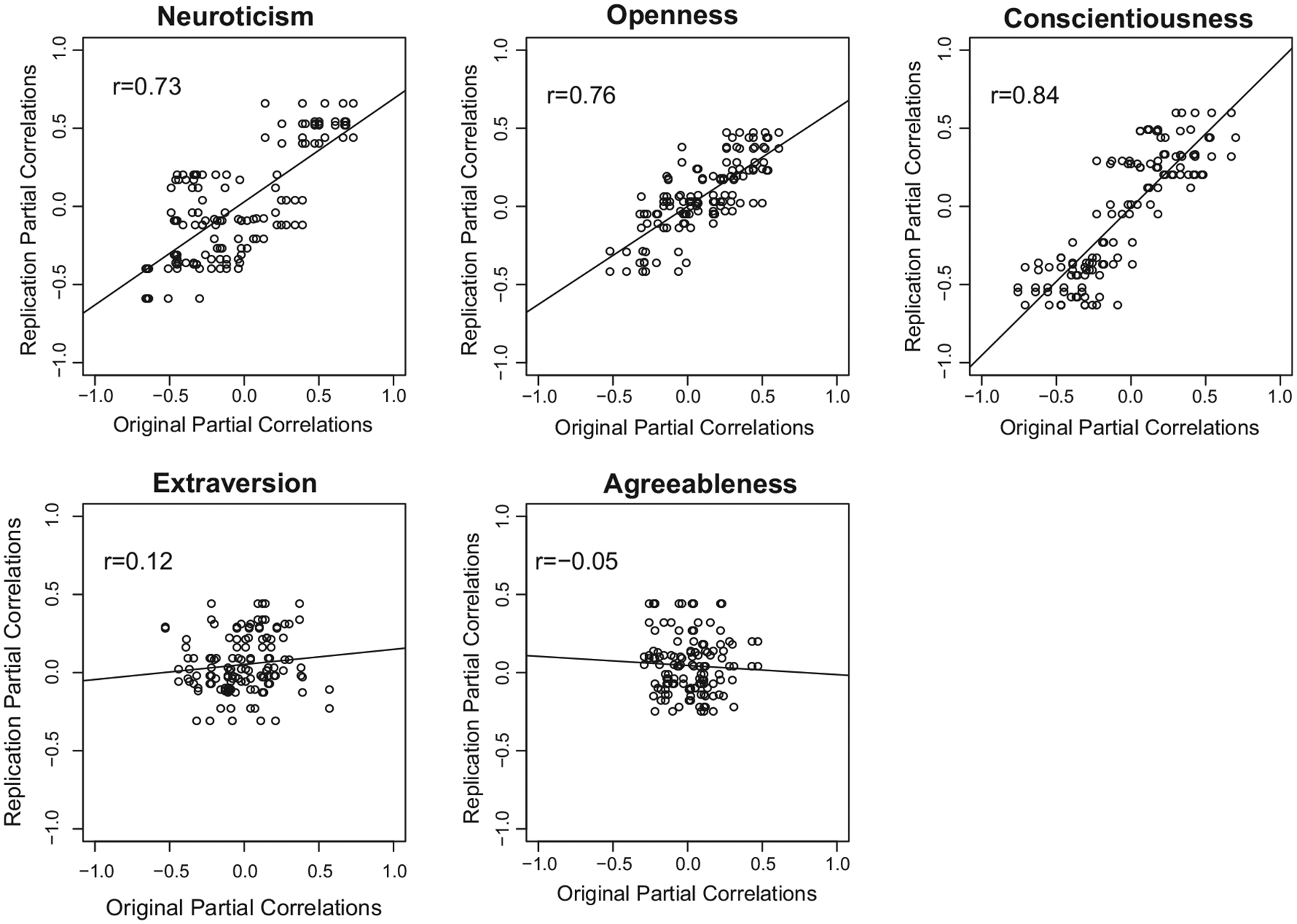
Replicability of correlations between state personality and 13 sociodemographic variables. Correlations of Samples 1–5 on *X*-axis. Correlations of SAPA2010 and SAPA2015 on *Y*-axis. The replication correlation (i.e., the correlation of correlation pairs) is listed for each trait. A simple linear regression line has been added to each trait for visual summarization.

To determine whether the two new samples replicated the correlations between the original samples and the sociodemographic variables, we correlated the correlations from the original samples with the correlations from the new samples. That is, within each Big Five trait, for each of the thirteen sociodemographic variables, we paired each of the five original correlations with each of the two new correlations, resulting in ten pairs of correlations per sociodemographic variable, for a total of 130 observation pairs for each trait (5 samples × 2 samples × 13 sociodemographic variables). Replication correlations were then found for these correlation pairs. The replication correlations differed greatly by trait (Fig. 2). Conscientiousness (*r* = .84), Openness (*r* = .76), and Neuroticism (*r* = .73) indicated high levels of replicability, whereas Extraversion (*r* = .12) and Agreeableness (*r* = −.05) showed little evidence for a consistent pattern of correlations with sociodemographic variables.

To ensure that the lack of replication for Extraversion and Agreeableness was not due to shared variance being removed due to partialling, replication correlations were found for Agreeableness and Extraversion without partialling other Big Five traits. Replication correlations improved for Extraversion (*r* = .19) and Agreeableness (*r* = .23), but the magnitude of these correlations was still well below that of the other three traits. The range of the 130 correlation pairs for each trait (that is, the minimum correlation subtracted from the maximum correlation) was also examined to determine if Extraversion or Agreeableness had a restricted range of correlations that could have been a limiting factor in the magnitude of its replication correlation. Extraversion and Agreeableness had the smallest ranges of correlations (1.1 and .76, respectively), compared to Conscientiousness (1.46), Neuroticism (1.39), and Openness (1.13). Thus, Agreeableness had a restricted range of correlations compared to the other traits, but the range of correlations for Extraversion was similar to that of Openness.

## 4. Discussion

State differences in Big Five personality may be used to predict important sociodemographic outcomes. These results replicate for three personality traits (Conscientiousness, Neuroticism, and Openness) across sampling methods, time periods, and personality inventories. In terms of agreement of scores across samples, same-trait correlations were highest for Neuroticism and Openness, but correlations for all five traits were positive, on average, indicating that state scores were somewhat robust. In Section 3.2, we showed that most of the personality variation was within states (small ICC1s), but between-state variation was still very reliable (large ICC2s). By the standard statistical criterion of “variance explained,” states appeared to be a poor candidate for grouping personality. The two replication samples were representative in terms of state populations and ethnicity within a state, were less representative of how educated states were, and were not representative of age groups within states. These representative analyses were similar to findings concerning the five samples from a previous study (Rentfrow et al., 2013).

Three differences across the samples (time period, participant recruitment related to research project, and personality inventory) differentially affected same-trait correlations. Different personality inventories were associated with some attenuation ($\bar{r}=.68$), which was minimal when adjusting for attenuation due to inventories’ reliabilities (Section 3.3.3). Same-trait correlations between samples in different projects, however, were substantially attenuated, and this attenuation was of the same magnitude whether samples were in the same time period ($\bar{r}=.38$), adjacent time periods ($\bar{r}=.41$), or in time periods separated by five years ($\bar{r}=.42$; Table 4). This result suggests that differences in time period were not related to attenuation over-and-above attenuation due to samples being from different projects and using different inventories. Although there was some attenuation associated with pairs of samples in adjacent time periods when projects and inventories were held constant ($\bar{r}=.68$; Table 4), this average same-trait correlation was of a similar magnitude to the estimated average reliability of inventories within the SAPA2015 sample ($\bar{r}=.75$; Section 3.3.3). These analyses lead to a conclusion that the rank order personality scores of U.S. states has been stable from 1999 to 2015. Because of this temporal stability and small ICC1 values in state-level personality, we recommend that researchers interested in utilizing our published state scores use the scores derived from combining the SAPA2010 and SAPA2015 data (Table 8 in the supplemental materials).

There are at least three reasons why Extraversion and Agreeableness were not found to have replicable correlations with sociodemographics. First, perhaps suboptimal sociodemographic measures were selected. Future studies might find that state-level Agreeableness and Extraversion have large, replicable correlations with sociodemographic measures not included in this study. While this is a possibility, several of the sociodemographic variables were selected based on previous research that showed moderate correlations with Agreeableness (crime and religiosity) and Extraversion (crime and business occupations). Second, the current study found that Agreeableness had a restricted range of correlations with the selected sociodemographic variables, which could indicate that correlations of a large magnitude are required to ensure replication at the state level. A third possible reason for lack of replicability is that lower-level facets within the traits differentially correlated with the sociodemographic variables. Rentfrow (2014) found that when Extraversion was split into two lower-level facets and correlated with six social indicators, the signs of the correlations were consistently opposite for the facets. Combining these kinds of opposing facets into a higher-level trait would result in the trait having smaller correlations and/or an inconsistent pattern of correlations with sociodemographic variables across samples. Future research should examine whether novel sociodemographics correlate more highly with Agreeableness and Extraversion, as well as whether facets within some traits differentially correlate with certain sociodemographic measures.

Researchers interested in the links between personality and geography may find new, important insights by examining personality constructs outside or at a lower level than the Big Five traits. Preliminary evidence in this paper indicated that state residence accounted for more total variance in cognitive ability and Liberalism than the Big Five. Additionally, for correlational analyses with sociodemographic variables, the level of a personality characteristic should match the level of a sociodemographic variable (Wittmann, 1988). That is, it may be more theoretically appropriate to correlate certain sociodemographic variables with facets instead of the Big Five.

In a similar way that personality constructs like the Big Five may be too broad in the study of geographical personality, geographical constructs may also be too broad. States may not be the optimal level for discovering geographical differences in personality. States share a common government, but they often cover large areas, and state lines can be arbitrary when grouping people by personality. For example, would we expect the personality of Paris, Illinois, a town of less than 10,000, to be more like Versailles, Indiana, or Chicago, Illinois? Both are roughly equidistant from Paris, but one is a town of less than 3000, and the other is the most populous city in the Midwest. Chicago is in the same state as Paris, so a state-level analysis would group both together and contrast them against another group comprised partly of Versailles and Indianapolis. It could be valuable for future research to compare the effect size of state aggregation to other regional groups, such as counties, metropolitan areas, cities, and neighborhoods.

Personality scores aggregated by geographical regions could prove to be invaluable to researchers interested in exploring macro-level relationships between patterns of human behavior, environment, and social outcomes. These scores would represent static measures for these regions like any other sociodemographic variable. For example, one could imagine a scenario in which the Extraversion of Illinois in 2020 would be a matter of public record in the same way that its population will be. Self-reported personality items have components of affect, behavior, cognition, and desire (Wilt & Revelle, 2015) and are presumed to reflect observable patterns in the real world. Having aggregate measures of these complex processes could help researchers to better understand mechanisms that drive important outcome patterns, such as rates of obesity, mental illness, crime, and well-being. In order for these scores to be trustworthy, however, they need to represent the population, aggregate individual variance, be reliably different, agree across samples, and have a consistent pattern of correlations with sociodemographic measures.

## Supplementary Material

Supplement

## Figures and Tables

**Table 1:** Summary description of samples.

Sample	Participant count	Personality inventory	Research project	Time period
Sample 1	612,140	44-item BFI	Gosling-Potter	1999–2005
Sample 2	507,987	44-item BFI	Gosling-Potter	2005–2009
Sample 3	145,307	10-item TIPI	Rentfrow-Potter	2002–2009
Sample 4	312,568	20-item NEO	MyPersonality	2008–2010
Sample 5	18,182	10-item TIPI	CCAP	2007–2008
SAPA2010	81,538	100-item BFFM	SAPA	2006–2010
SAPA2015	134,858	100-item BFFM	SAPA	2010–2015

**Table 2:** Average participants per state (*k*), mean ICC1 and mean ICC2 across the Big Five traits, for each sample. ICC1 is the percent of total personality variance accounted for by state residence. ICC2 is the group mean reliability of state scores.

Sample	*k*	ICC1 (Mean)	ICC2 (Mean)
Sample1	12,493	0.0018	0.95
Sample2	10,367	0.0020	0.96
Sample3	2965	0.0004	0.82
Sample4	6379	0.0013	0.94
Sample5	382	0.0001	0.52
SAPA2010	1598	0.0027	0.79
SAPA2015	2577	0.0030	0.88
SAPA2015 (Simulation)	2577	0.0000	−0.05
SAPA2015 (Liberalism)	459	0.0137	0.86
SAPA2015 (Cog. Abil.)	2602	0.0122	0.97

**Table 3:** Standard deviation and range of unstandardized personality scores at the individual and state levels in the SAPA2015 sample.

Personality trait	Mean	Invid. SD	State SD	Indiv. range	State range
Conscientiousness	4.20	1.02	0.08	5 (1–6)	0.42 (4.04–4.45)
Agreeableness	4.69	0.88	0.06	5 (1–6)	0.30 (4.50–4.80)
Emotional Stability	3.58	1.18	0.07	5 (1–6)	0.29 (3.44–3.72)
Intellect	4.55	0.88	0.08	5 (1–6)	0.37 (4.38–4.75)
Extraversion	3.84	1.18	0.08	5 (1–6)	0.43 (3.54–3.97)
Liberalism	3.47	1.55	0.26	5 (1–6)	1.44 (2.87–4.31)

**Table 4:** Census-weighted same-trait correlations of state-level personality, across samples. Sample pairs grouped by similarities and differences in personality inventories, research projects, and time period.

Sample pair group^a^	Sample pair	Consc.	Agree.	Neuro.	Open.	Extra.	Mean^b^
Inv:S, Proj:S, Time:A	Sample 1 – Sample 2	0.72	0.76	0.80	0.85	0.70	0.77
	SAPA2010 – SAPA2015	0.41	0.47	0.64	0.63	0.65	0.57
	Mean^b^	0.59	0.64	0.73	0.76	0.68	0.68
Inv:S, Proj:D, Time:S	Sample 3 – Sample 5	0.23	0.05	0.47	0.34	−0.20	0.19
Inv:D, Proj:D, Time:S	Sample 2 – Sample 3	0.45	0.49	0.66	0.62	0.59	0.57
	Sample 2 – Sample 4	0.61	0.28	0.67	0.85	0.21	0.57
	Sample 2 – Sample 5	0.31	0.31	0.45	0.68	−0.11	0.35
	Sample 3 – Sample 4	0.14	0.37	0.64	0.67	0.12	0.41
	Sample 4 – Sample 5	0.27	0.19	0.63	0.65	0.46	0.46
	Sample 2 – SAPA2010	0.27	0.19	0.41	0.48	0.20	0.32
	Sample 3 – SAPA2010	0.12	0.15	0.48	0.19	0.36	0.27
	Sample 4 – SAPA2010	0.21	−0.16	0.53	0.29	0.35	0.25
	Sample 5 – SAPA2010	0.11	−0.23	0.40	0.27	0.17	0.15
	Mean^b^	0.29	0.18	0.55	0.56	0.27	0.38
Inv:D, Proj:D, Time:A	Sample 1 – Sample 3	0.37	0.53	0.77	0.70	0.56	0.60
	Sample 1 – Sample 4	0.70	0.62	0.74	0.72	0.20	0.62
	Sample 1 – Sample 5	0.30	0.26	0.54	0.53	−0.06	0.33
	Sample 1 – SAPA2010	0.26	−0.02	0.53	0.43	0.27	0.30
	Sample 2 – SAPA2015	0.47	0.41	0.66	0.36	0.25	0.44
	Sample 3 – SAPA2015	0.37	0.22	0.59	0.09	0.21	0.31
	Sample 4 – SAPA2015	0.41	−0.16	0.72	0.18	0.37	0.34
	Sample 5 – SAPA2015	0.43	0.11	0.49	0.22	0.09	0.28
	Mean^b^	0.43	0.26	0.64	0.43	0.24	0.41
Inv:D, Proj:D, Time:N	Sample 1 – SAPA2015	0.37	0.11	0.76	0.43	0.28	0.42

a Inv = Personality inventory; Proj = Research project; Time = Time period; S = Same; A = Adjacent; D = Different; N = Non-adjacent.

b A mean was found by converting correlations to *z*-scores, finding the mean, and converting the mean *z*-score to a correlation.

**Table 5:** Mean census-weighted bootstrapped same-trait correlations of state-level personality, across four inventories within the SAPA2015 sample. Based on 1000 iterations of splitting the sample into two random halves and correlating the state scores from each half. Below the diagonal are raw correlations. On the diagonal are mean reliabilities. Above the diagonal are correlations adjusted for the reliabilities.

Personality trait	Inventory^a^	BFFM	NEO	SPI	HEX
Conscientiousness	BFFM	0.83	0.97	0.96	0.97
	NEO	0.81	0.84	0.96	0.98
	SPI	0.81	0.81	0.85	0.90
	HEXACO	0.80	0.81	0.75	0.82
Agreeableness	BFFM	0.75	0.82	0.95	0.14
	NEO	0.60	0.71	0.90	0.29
	SPI	0.65	0.59	0.63	0.19
	HEXACO	0.10	0.21	0.13	0.73
Neuroticism	BFFM	0.74	0.97	0.98	0.97
	NEO	0.70	0.71	0.92	0.91
	SPI	0.70	0.64	0.69	1.00
	HEXACO	0.60	0.55	0.60	0.53
Openness	BFFM	0.80	0.94	0.99	0.94
	NEO	0.75	0.79	0.98	0.98
	SPI	0.76	0.75	0.74	0.96
	HEXACO	0.73	0.76	0.72	0.78
Extraversion	BFFM	0.72	1.00	0.95	0.97
	NEO	0.74	0.76	0.97	0.99
	SPI	0.70	0.74	0.76	0.92
	HEXACO	0.70	0.73	0.68	0.72

a Inventories were from the International Personality Item Pool.

## Footnotes

1 ICC1 was calculated for Samples 1–5 by utilizing a derivation of Eq. (1), average number of participants (*k*), and ICC2.

2 Samples also differed in their number of participants, and samples with less participants had lower group mean reliabilities. Correlations adjusted for attenuation due to reliability are available in Fig. 1 of the supplemental materials.

3 Several same-trait correlations in Agreeableness were very small, which appeared to be caused by the HEXACO Agreeableness scale (Table 5). Due to the low average same-trait correlation of the Big Five measures of Agreeableness with HEXACO Agreeableness ($\bar{r}=.15$), we calculated the correlations of the Big Five Agreeableness measures with HEXACO Honesty-Humility and found the average correlation to be positive and moderate ($\bar{r}=.41$). Thus, for state-level personality, the Big Five Agreeableness construct appeared to be more closely related to HEXACO Honesty-Humility than HEXACO Agreeableness.
