# Supplementary material for: The personality of U.S. states: Stability from 1999 to 2015
Source: J Res Pers. Author manuscript; Available in PMC 2020 Aug 21. (PMC7441846; doi:10.1016/j.jrp.2016.06.022)
Supplement: Supplement [file NIHMS1564617-supplement-Supplement.pdf]

Supplemental material to: The Personality U.S. States: Stability from 1999 to 2015

Lorien G. Elleman, David M. Condon, Sarah E. Russin, and William Revelle

Figure 1: Census-weighted same-trait correlations of state-level personality, adjusted for ICC2 attenuation. Color-coded for size and sign of correlation. Upper triangle shows correlations with decimal removed. Diagonal is the group mean reliability of the trait (ICC2) for a given sample.

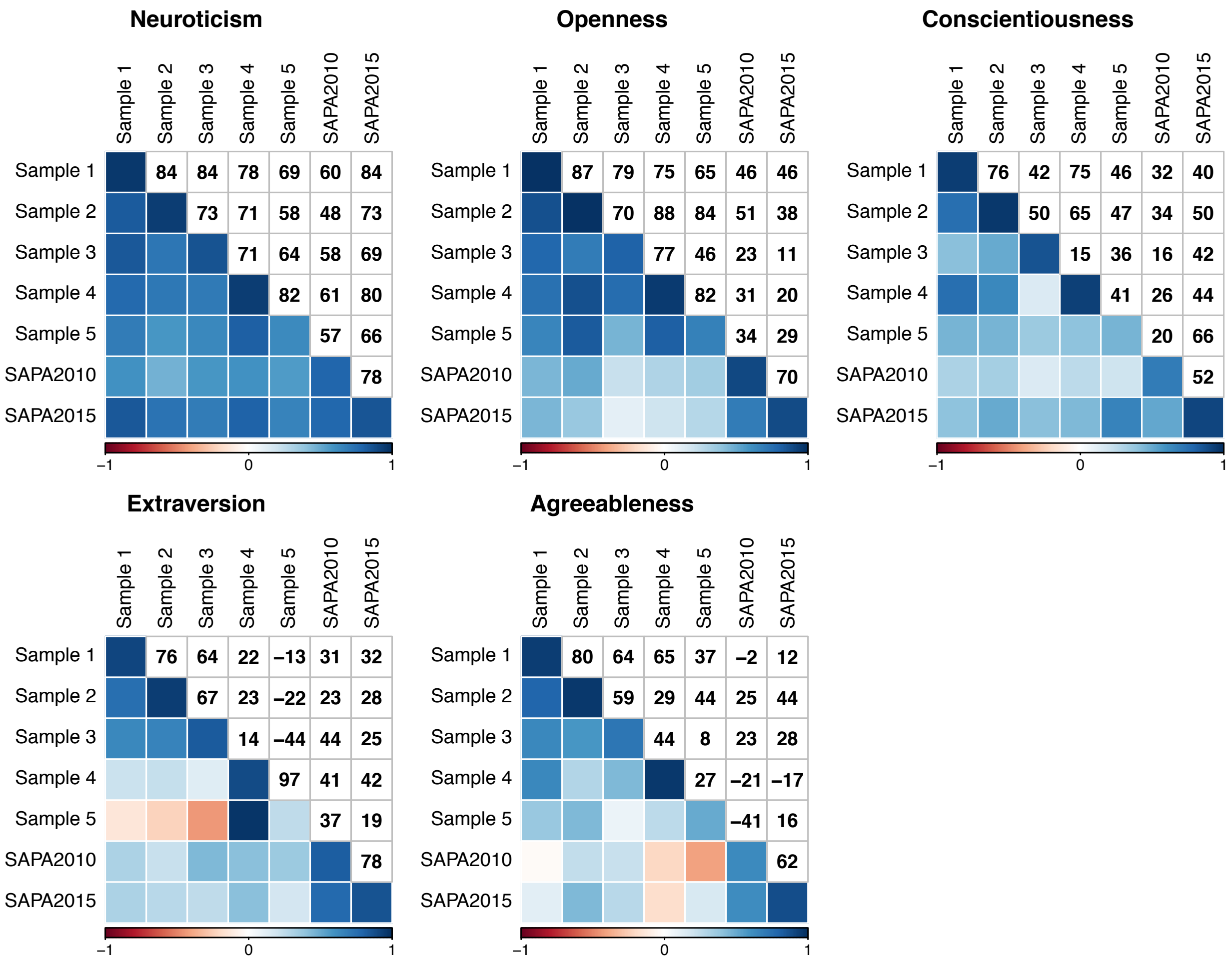

Sociodemographic Variable	Sample 1	Sample 2	Sample 3	Sample 4	Sample 5	SAPA2010	SAPA2015
Violent Crime	0.43	0.22	0.23	0.42	0.33	0.20	0.33
Property Crime	0.53	0.52	0.20	0.70	0.23	0.20	0.44
Cancer Deaths	-0.23	-0.13	-0.01	-0.06	0.18	0.29	-0.05
Heart Disease Deaths	0.18	0.17	0.06	0.33	0.06	0.25	0.48
Arts... and Media Jobs	-0.39	-0.30	-0.28	-0.62	-0.26	-0.36	-0.41
Computer and Math. Science Jobs	-0.31	-0.37	-0.21	-0.40	-0.36	-0.44	-0.58
Healthcare Pract. and Technical Jobs	0.11	0.18	0.12	0.40	0.12	0.49	0.12
Business and Financial Jobs	-0.26	-0.47	-0.23	-0.47	-0.09	-0.33	-0.63
Patents Issued	-0.64	-0.56	-0.33	-0.76	-0.45	-0.52	-0.55
Percent Liberal	-0.55	-0.47	-0.31	-0.71	-0.31	-0.39	-0.63
Religion Important	0.54	0.43	0.30	0.67	0.34	0.32	0.60
Well-being	-0.18	-0.19	0.01	-0.39	-0.12	-0.37	-0.23
Community Recognition	0.04	0.13	-0.02	0.00	-0.14	0.01	0.27

Table 1: Census-weighted partial correlations for state-level Conscientiousness and 13 sociodemographic variables, across samples. Controlling for the other Big Five traits.

Sociodemographic Variable	Sample 1	Sample 2	Sample 3	Sample 4	Sample 5	SAPA2010	SAPA2015
Violent Crime	0.02	0.01	0.01	-0.15	-0.18	-0.11	-0.18
Property Crime	0.09	-0.22	0.17	-0.10	0.11	-0.07	-0.25
Cancer Deaths	0.05	0.02	-0.20	0.05	0.13	-0.10	0.13
Heart Disease Deaths	0.02	0.24	0.12	-0.23	0.17	0.14	-0.15
Arts... and Media Jobs	-0.09	-0.05	-0.29	0.03	-0.19	0.05	0.10
Computer and Math. Science Jobs	0.03	0.22	-0.06	-0.22	0.04	0.27	0.44
Healthcare Pract. and Technical Jobs	0.22	0.10	0.11	0.10	0.30	-0.05	0.18
Business and Financial Jobs	-0.04	0.23	-0.23	-0.22	-0.26	0.09	0.44
Patents Issued	0.47	0.28	-0.07	0.43	0.04	0.04	0.20
Percent Liberal	0.07	0.15	-0.26	-0.16	-0.11	0.11	0.32
Religion Important	0.08	0.12	0.31	-0.06	0.11	0.04	-0.22
Well-being	-0.09	-0.13	-0.03	0.08	-0.14	-0.07	-0.04
Community Recognition	-0.13	-0.15	0.21	0.09	0.11	-0.01	-0.14

Table 2: Census-weighted partial correlations for state-level Agreeableness and 13 sociodemographic variables, across samples. Controlling for the other Big Five traits.

Sociodemographic Variable	Sample 1	Sample 2	Sample 3	Sample 4	Sample 5	SAPA2010	SAPA2015
Violent Crime	0.13	0.08	0.06	-0.04	-0.20	-0.21	-0.08
Property Crime	-0.12	-0.22	-0.04	-0.16	-0.45	-0.40	-0.34
Cancer Deaths	0.39	0.25	0.50	0.47	0.41	0.40	0.53
Heart Disease Deaths	0.66	0.73	0.54	0.39	0.14	0.44	0.66
Arts... and Media Jobs	-0.35	-0.13	-0.49	-0.31	0.21	0.12	-0.04
Computer and Math. Science Jobs	-0.32	-0.32	-0.34	-0.45	-0.12	0.20	-0.37
Healthcare Pract. and Technical Jobs	0.61	0.50	0.67	0.68	0.47	0.52	0.54
Business and Financial Jobs	-0.34	-0.39	-0.44	-0.46	-0.03	0.17	-0.36
Patents Issued	-0.45	-0.47	-0.46	-0.26	-0.02	-0.09	-0.31
Percent Liberal	-0.28	-0.19	-0.33	-0.41	0.22	0.20	-0.12
Religion Important	0.39	0.34	0.28	0.25	-0.28	-0.12	0.04
Well-being	-0.66	-0.65	-0.64	-0.51	-0.30	-0.40	-0.59
Community Recognition	-0.15	0.02	-0.18	-0.02	-0.16	-0.27	-0.09

Table 3: Census-weighted partial correlations for state-level Neuroticism and 13 sociodemographic variables, across samples. Controlling for the other Big Five traits.

Sociodemographic Variable	Sample 1	Sample 2	Sample 3	Sample 4	Sample 5	SAPA2010	SAPA2015
Violent Crime	0.29	0.37	0.33	0.17	-0.14	0.03	0.19
Property Crime	-0.06	0.02	-0.14	-0.16	-0.31	-0.14	0.06
Cancer Deaths	-0.12	-0.16	0.06	0.22	0.02	0.00	0.03
Heart Disease Deaths	-0.27	0.17	-0.03	-0.02	-0.20	-0.11	-0.05
Arts... and Media Jobs	0.53	0.44	0.41	0.30	0.54	0.44	0.23
Computer and Math. Science Jobs	0.31	0.10	0.22	-0.13	0.24	0.18	0.17
Healthcare Pract. and Technical Jobs	-0.29	-0.32	-0.21	-0.01	-0.29	-0.36	-0.05
Business and Financial Jobs	0.51	0.33	0.45	-0.04	0.26	0.38	0.28
Patents Issued	0.50	0.07	0.06	0.35	0.44	0.24	0.02
Percent Liberal	0.61	0.35	0.44	0.26	0.52	0.47	0.37
Religion Important	-0.41	-0.06	-0.28	-0.30	-0.52	-0.42	-0.29
Well-being	0.30	0.07	0.25	-0.05	0.17	0.07	-0.03
Community Recognition	0.01	-0.03	0.18	0.18	0.17	-0.05	0.03

Table 4: Census-weighted partial correlations for state-level Openness and 13 sociodemographic variables, across samples. Controlling for the other Big Five traits.

Sociodemographic Variable	Sample 1	Sample 2	Sample 3	Sample 4	Sample 5	SAPA2010	SAPA2015
Violent Crime	-0.02	0.04	-0.20	0.30	0.27	0.08	0.31
Property Crime	-0.23	-0.08	-0.32	0.21	0.11	-0.31	-0.02
Cancer Deaths	0.03	0.15	0.26	0.15	-0.10	0.22	0.01
Heart Disease Deaths	-0.22	0.16	0.20	0.38	-0.11	0.03	-0.02
Arts... and Media Jobs	0.18	0.01	0.12	-0.39	-0.05	0.21	0.16
Computer and Math. Science Jobs	-0.04	-0.19	-0.08	-0.38	0.04	-0.04	0.09
Healthcare Pract. and Technical Jobs	-0.11	0.13	0.12	0.39	-0.06	-0.03	-0.13
Business and Financial Jobs	0.37	0.14	0.12	-0.22	0.09	0.34	0.44
Patents Issued	-0.09	-0.37	-0.02	-0.44	0.01	0.02	-0.06
Percent Liberal	0.22	-0.05	0.10	-0.53	0.03	0.28	0.29
Religion Important	-0.16	0.04	-0.09	0.57	0.07	-0.23	-0.11
Well-being	0.16	-0.23	-0.22	-0.34	0.17	-0.07	0.09
Community Recognition	-0.13	-0.31	-0.10	-0.11	-0.10	-0.12	-0.10

Table 5: Census-weighted partial correlations for state-level Extraversion and 13 sociodemographic variables, across samples. Controlling for the other Big Five traits.

State	Sample Size	Conscientiousness	Agreeableness	Stability*	Intellect†	Extraversion
Alabama	644	4.12	4.61	3.47	4.64	3.74
Alaska	556	4.03	4.63	3.65	4.66	3.80
Arizona	865	4.10	4.60	3.58	4.67	3.83
Arkansas	577	4.15	4.69	3.55	4.60	3.83
California	9712	4.10	4.66	3.63	4.61	3.92
Colorado	1096	4.12	4.63	3.64	4.67	3.81
Connecticut	988	4.12	4.64	3.55	4.65	3.92
Delaware	591	4.00	4.57	3.56	4.38	4.02
Florida	2935	4.18	4.66	3.59	4.66	3.89
Georgia	2414	4.13	4.69	3.63	4.60	4.00
Hawaii	292	4.17	4.64	3.69	4.53	3.84
Idaho	340	4.15	4.67	3.59	4.68	3.75
Illinois	5521	4.16	4.73	3.62	4.57	4.01
Indiana	1706	4.22	4.69	3.61	4.58	3.90
Iowa	982	4.10	4.67	3.64	4.53	3.91
Kansas	808	4.11	4.68	3.62	4.60	3.94
Kentucky	819	4.12	4.69	3.59	4.60	3.94
Louisiana	2029	4.21	4.70	3.67	4.42	4.01
Maine	356	4.21	4.67	3.54	4.64	3.85
Maryland	1773	4.13	4.71	3.59	4.58	3.92
Massachusetts	1936	4.12	4.66	3.50	4.57	3.90
Michigan	2550	4.16	4.65	3.56	4.66	3.90
Minnesota	2104	4.10	4.67	3.60	4.53	3.94
Mississippi	604	4.20	4.72	3.56	4.56	3.92
Missouri	1611	4.14	4.66	3.56	4.59	3.91
Montana	243	4.16	4.76	3.60	4.71	3.85
Nebraska	580	4.16	4.64	3.59	4.50	3.92
Nevada	274	4.08	4.64	3.64	4.65	3.83
New Hampshire	389	4.15	4.63	3.49	4.63	3.86
New Jersey	2495	4.15	4.67	3.58	4.61	4.00
New Mexico	1199	4.26	4.73	3.70	4.58	3.90
New York	4941	4.15	4.66	3.51	4.65	3.93
North Carolina	1454	4.17	4.65	3.53	4.57	3.81
North Dakota	190	4.29	4.61	3.53	4.57	3.80
Ohio	3597	4.19	4.70	3.54	4.56	3.92
Oklahoma	771	4.14	4.65	3.56	4.61	3.80
Oregon	1203	4.07	4.64	3.70	4.62	3.89
Pennsylvania	4760	4.12	4.66	3.48	4.51	3.96
Rhode Island	422	4.10	4.73	3.67	4.62	4.06
South Carolina	1008	4.14	4.73	3.70	4.49	4.04
South Dakota	172	4.27	4.66	3.80	4.65	3.98
Tennessee	1133	4.21	4.66	3.62	4.59	3.84
Texas	4663	4.13	4.65	3.59	4.61	3.89
Utah	487	4.10	4.67	3.68	4.61	3.88
Vermont	161	4.09	4.78	3.59	4.74	3.92
Virginia	2785	4.14	4.72	3.57	4.55	3.94
Washington	1739	4.13	4.65	3.62	4.68	3.81
West Virginia	384	4.16	4.62	3.60	4.58	3.78
Wisconsin	2379	4.11	4.64	3.62	4.50	3.92
Wyoming	126	4.19	4.66	3.78	4.63	3.82
District of Columbia	174	4.17	4.63	3.57	4.78	3.94

*Emotional Stability. To find a Neuroticism score, use the following formula: 7 - Emotional Stability score.
†Intellect state scores were highly correlated with Openness state scores from other inventories ($rs \geq .9$).

Table 6: SAPA2010 sample sizes and aggregate unstandardized state personality scores (*range* = 1-6) for the 100-item IPIP Big Five Factor Markers (BFFM) scale.

State	Sample Size	Conscientiousness	Agreeableness	Stability*	Intellect†	Extraversion
Alabama	1474	4.29	4.73	3.60	4.62	3.79
Alaska	520	4.09	4.50	3.66	4.60	3.71
Arizona	2096	4.14	4.65	3.59	4.56	3.76
Arkansas	759	4.19	4.69	3.53	4.63	3.75
California	15160	4.16	4.66	3.62	4.55	3.82
Colorado	1557	4.17	4.61	3.70	4.69	3.72
Connecticut	1079	4.09	4.61	3.47	4.65	3.72
Delaware	1646	4.10	4.56	3.60	4.38	3.93
Florida	6692	4.26	4.70	3.62	4.58	3.87
Georgia	4508	4.30	4.73	3.62	4.56	3.91
Hawaii	426	4.15	4.64	3.72	4.56	3.80
Idaho	540	4.13	4.63	3.59	4.64	3.64
Illinois	9789	4.21	4.74	3.61	4.52	3.95
Indiana	2336	4.12	4.61	3.49	4.56	3.80
Iowa	1280	4.12	4.64	3.65	4.41	3.85
Kansas	1001	4.15	4.58	3.62	4.61	3.74
Kentucky	1561	4.14	4.59	3.57	4.45	3.77
Louisiana	2262	4.30	4.73	3.58	4.45	3.92
Maine	491	4.14	4.63	3.55	4.59	3.76
Maryland	2389	4.17	4.68	3.57	4.60	3.84
Massachusetts	2688	4.11	4.70	3.57	4.60	3.81
Michigan	6093	4.26	4.74	3.51	4.51	3.78
Minnesota	3538	4.16	4.67	3.59	4.50	3.86
Mississippi	910	4.39	4.70	3.62	4.50	3.87
Missouri	2147	4.16	4.67	3.55	4.58	3.78
Montana	467	4.27	4.65	3.69	4.67	3.88
Nebraska	1090	4.22	4.69	3.58	4.45	3.76
Nevada	584	4.29	4.59	3.70	4.65	3.76
New Hampshire	511	4.23	4.72	3.60	4.67	3.80
New Jersey	2714	4.16	4.69	3.53	4.58	3.80
New Mexico	2065	4.27	4.72	3.63	4.61	3.88
New York	7578	4.18	4.68	3.46	4.57	3.86
North Carolina	3038	4.27	4.69	3.59	4.55	3.81
North Dakota	235	4.04	4.68	3.64	4.49	3.76
Ohio	5426	4.25	4.71	3.55	4.55	3.85
Oklahoma	1416	4.22	4.61	3.54	4.60	3.84
Oregon	1993	4.12	4.67	3.64	4.63	3.79
Pennsylvania	6817	4.14	4.66	3.44	4.48	3.88
Rhode Island	695	4.19	4.75	3.56	4.58	3.90
South Carolina	2264	4.45	4.80	3.60	4.50	3.90
South Dakota	299	4.32	4.73	3.68	4.51	3.84
Tennessee	1833	4.23	4.73	3.57	4.59	3.76
Texas	9013	4.20	4.65	3.61	4.54	3.84
Utah	1085	4.17	4.69	3.71	4.57	3.68
Vermont	273	4.24	4.73	3.52	4.61	3.69
Virginia	5182	4.26	4.79	3.63	4.52	3.87
Washington	3547	4.10	4.62	3.60	4.58	3.72
West Virginia	441	4.19	4.75	3.49	4.70	3.72
Wisconsin	2920	4.15	4.68	3.57	4.50	3.81
Wyoming	180	4.36	4.63	3.49	4.64	3.54
District of Columbia	250	4.08	4.60	3.64	4.75	3.97

*Emotional Stability. To find a Neuroticism score, use the following formula: 7 - Emotional Stability score.
†Intellect state scores were highly correlated with Openness state scores from other inventories ($rs \geq .9$).

Table 7: SAPA2015 sample sizes and aggregate unstandardized state personality scores ($range$ = 1-6) for the 100-item IPIP Big Five Factor Markers (BFFM) scale.

State	Sample Size	Conscientiousness	Agreeableness	Stability*	Intellect†	Extraversion
Alabama	2118	4.23	4.69	3.56	4.63	3.78
Alaska	1076	4.06	4.57	3.66	4.63	3.76
Arizona	2961	4.12	4.63	3.59	4.59	3.78
Arkansas	1336	4.17	4.69	3.54	4.62	3.78
California	24872	4.14	4.66	3.62	4.57	3.86
Colorado	2653	4.15	4.62	3.68	4.68	3.76
Connecticut	2067	4.10	4.62	3.51	4.65	3.82
Delaware	2237	4.08	4.56	3.59	4.38	3.96
Florida	9627	4.24	4.69	3.61	4.60	3.88
Georgia	6922	4.24	4.72	3.62	4.57	3.94
Hawaii	718	4.16	4.64	3.71	4.55	3.82
Idaho	880	4.14	4.65	3.59	4.65	3.69
Illinois	15310	4.20	4.74	3.61	4.54	3.97
Indiana	4042	4.16	4.64	3.54	4.57	3.84
Iowa	2262	4.11	4.65	3.65	4.46	3.88
Kansas	1809	4.13	4.62	3.62	4.61	3.83
Kentucky	2380	4.14	4.63	3.58	4.50	3.83
Louisiana	4291	4.26	4.72	3.62	4.44	3.96
Maine	847	4.17	4.65	3.55	4.62	3.80
Maryland	4162	4.15	4.69	3.58	4.59	3.88
Massachusetts	4624	4.11	4.68	3.54	4.59	3.85
Michigan	8643	4.23	4.71	3.53	4.55	3.81
Minnesota	5642	4.14	4.67	3.59	4.51	3.89
Mississippi	1514	4.31	4.71	3.60	4.52	3.89
Missouri	3758	4.15	4.67	3.56	4.58	3.84
Montana	710	4.23	4.69	3.66	4.68	3.87
Nebraska	1670	4.19	4.67	3.59	4.47	3.82
Nevada	858	4.22	4.61	3.68	4.65	3.78
New Hampshire	900	4.19	4.68	3.55	4.65	3.82
New Jersey	5209	4.15	4.68	3.55	4.60	3.89
New Mexico	3264	4.27	4.72	3.66	4.60	3.88
New York	12519	4.17	4.67	3.48	4.60	3.89
North Carolina	4492	4.24	4.68	3.57	4.56	3.81
North Dakota	425	4.15	4.65	3.59	4.52	3.78
Ohio	9023	4.22	4.71	3.54	4.55	3.88
Oklahoma	2187	4.19	4.62	3.55	4.60	3.83
Oregon	3196	4.10	4.66	3.66	4.63	3.83
Pennsylvania	11577	4.13	4.66	3.45	4.49	3.91
Rhode Island	1117	4.15	4.74	3.60	4.59	3.96
South Carolina	3272	4.35	4.78	3.64	4.50	3.95
South Dakota	471	4.30	4.70	3.73	4.56	3.89
Tennessee	2966	4.22	4.70	3.59	4.59	3.79
Texas	13676	4.18	4.65	3.60	4.57	3.86
Utah	1572	4.15	4.68	3.70	4.58	3.75
Vermont	434	4.19	4.75	3.55	4.66	3.78
Virginia	7967	4.22	4.77	3.61	4.53	3.90
Washington	5286	4.11	4.63	3.61	4.61	3.75
West Virginia	825	4.18	4.69	3.54	4.64	3.75
Wisconsin	5299	4.13	4.66	3.59	4.50	3.86
Wyoming	306	4.29	4.65	3.62	4.64	3.66
District of Columbia	424	4.12	4.61	3.61	4.76	3.96

*Emotional Stability. To find a Neuroticism score, use the following formula: 7 - Emotional Stability score.
†Intellect state scores were highly correlated with Openness state scores from other inventories ($rs \geq .9$).

Table 8: Combined SAPA (SAPA2010 + SAPA2015) sample sizes and aggregate unstandardized state personality scores ($range$ = 1-6) for the 100-item IPIP Big Five Factor Markers (BFFM) scale.
